# Nucleic Acid Extraction and Sequencing from Low-Biomass Synthetic Mars Analog Soils for *In Situ* Life Detection

**DOI:** 10.1089/ast.2018.1929

**Published:** 2019-08-22

**Authors:** Angel Mojarro, Julie Hachey, Ryan Bailey, Mark Brown, Robert Doebler, Gary Ruvkun, Maria T. Zuber, Christopher E. Carr

**Affiliations:** ^1^ Department of Earth, Atmospheric and Planetary Sciences, Massachusetts Institute of Technology, Cambridge, Massachusetts.; ^2^ Readcoor, Cambridge, Massachusetts.; ^3^ Claremont Biosolutions, LLC, Upland, California.; ^4^ Department of Molecular Biology, Massachusetts General Hospital, Boston, Massachusetts.

**Keywords:** Life-detection instruments, Nucleic acids, Origins of life, Panspermia, Mars.

## Abstract

Recent studies regarding the origins of life and Mars-Earth meteorite transfer simulations suggest that biological informational polymers, such as nucleic acids (DNA and RNA), have the potential to provide unambiguous evidence of life on Mars. To this end, we are developing a metagenomics-based life-detection instrument which integrates nucleic acid extraction and nanopore sequencing: the Search for Extra-Terrestrial Genomes (SETG). Our goal is to isolate and sequence nucleic acids from extant or preserved life on Mars in order to determine if a particular genetic sequence (1) is distantly related to life on Earth, indicating a shared ancestry due to lithological exchange, or (2) is unrelated to life on Earth, suggesting a convergent origins of life on Mars. In this study, we validate prior work on nucleic acid extraction from cells deposited in Mars analog soils down to microbial concentrations (*i.e.,* 10^4^ cells in 50 mg of soil) observed in the driest and coldest regions on Earth. In addition, we report low-input nanopore sequencing results from 2 pg of purified *Bacillus subtilis* spore DNA simulating ideal extraction yields equivalent to 1 ppb life-detection sensitivity. We achieve this by employing carrier sequencing, a method of sequencing sub-nanogram DNA in the background of a genomic carrier. After filtering of carrier, low-quality, and low-complexity reads we detected 5 *B. subtilis* reads, 18 contamination reads (including *Homo sapiens*), and 6 high-quality noise reads believed to be sequencing artifacts.

## 1. Introduction

Major strides in understanding the origins of life and meteorite transfer simulations support the notion that life on Mars, if it ever existed, may share a common genesis or perhaps share a common ancestry with life on Earth. Namely, analogous prebiotic environments (Johnson *et al.,* 2008; Morris *et al.,* 2010; Stoker *et al.,* 2010; Grotzinger *et al.,* 2014; Ranjan and Sasselov, 2017; Ranjan *et al.,* 2017), molecular feedstocks (*e.g.,* hydrogen cyanide) (Brack and Pillinger, 1998; Parker *et al.,* 2011; Adcock *et al.,* 2013), and plausible abiotic reactive pathways predicted on Earth and applicable on Mars may have resulted in parallel origin events in accordance with the RNA-world hypothesis (Powner *et al.,* 2009, 2010; McKay, 2010; Ritson and Sutherland, 2012; Benner and Kim, 2015; Patel *et al.,* 2015; Stairs *et al.,* 2017). This hypothesis suggests that past or present martian life may have utilized known building blocks (*e.g.,* nucleic acids, sugars, amino acids) and closely resembled life as we know it. Moreover, nonsterilizing lithological exchange between Mars and Earth from impact ejecta produced during the presumed Late Heavy Bombardment period (Gomes *et al.,* 2005; Boehnke and Harrison, 2016) may have transported viable microbes between planets (Weiss, 2000; Shuster, 2005; Horneck *et al.,* 2008; Abramov and Mojzsis, 2009), resulting in ancestrally related life (Isenbarger *et al.,* 2008).

To test these hypotheses, we are developing the Search for Extra-Terrestrial Genomes (SETG) life-detection instrument for *in situ* extraction and nanopore sequencing of nucleic acids (Carr *et al.,* 2016, 2017) from extant or preserved life on Mars. Assuming a convergent adoption of nucleic acids as the unitary solution for genetic information storage and transmission (*e.g.,* DNA or RNA), long-read nanopore sequencing could be capable of detecting nonstandard nucleic acids (Carr, 2016; Carr *et al.,* 2017) possibly endemic to life on Mars (Ranjan *et al.,* 2017). Furthermore, long-read sequencing is of particular significance for taxonomic identification at or below the species level (Greninger *et al.,* 2015; Quick *et al.,* 2015; Benítez-Páez *et al.,* 2016; Brown *et al.,* 2017), which would permit the detection of microbial forward contamination. For instance, in the case of ancestrally related life, comparing sequence data detected on Mars to conserved genes (Makarova *et al.,* 1999; Harris, 2003) on Earth (*i.e.,* the ribosome) (Woese *et al.,* 1975) could unambiguously discriminate forward contamination from a true life detection (Isenbarger *et al.,* 2008). Conversely, detecting a genetic sequence unlike anything found on Earth (including nonstandard bases) could signify a second genesis and perhaps indicate that nucleic acid–based life is common.

SETG operates by first extracting and isolating nucleic acids (DNA or RNA) from cells in solid or liquid samples using a modified Claremont BioSolutions solid-phase Purelyse bacterial genomic DNA extraction kit. Prior studies have utilized OmniLyse, which is Purelyse without extraction buffers, to lyse cells in an RNA extraction module aboard the International Space Station (Parra *et al.,* 2017). Long-read sequencing is then conducted using the Oxford Nanopore Technologies MinION, which sequences nucleic acids via ionic current monitoring (Lu *et al.,* 2016) and has been validated to sequence DNA in microgravity (McIntyre *et al.,* 2016; Castro-Wallace *et al.,* 2017), lunar and martian gravity (Carr/Zuber, unpublished data), and under simulated martian temperature and pressure (Carr *et al.*, 2019). Readers interested in the current status of SETG, including technology development, demonstration, and subsystem development, should refer to the works of Carr *et al.* (2017) and Bhattaru (2018).

Our goal for SETG is to be capable of analyzing a variety of environmental samples relevant to the search for life (related or otherwise to life on Earth) on Mars. However, complex soils, especially those containing iron oxides found on Mars (Bell *et al.,* 2000), greatly inhibit nucleic acid extraction from cells due to competitive adsorption onto mineral surfaces (Jiang *et al.,* 2012; Hurt *et al.,* 2014) or destruction due to hydroxyl radicals (Imlay and Linn, 1988; Gates, 2009) during cell lysis (Mojarro *et al.,* 2017b). Additional inhibition may occur in the presence of phyllosilicates (Greaves and Wilson, 1969; Melzak *et al.,* 1996; Trevors, 1996) and salts (Henneberger *et al.,* 2006) due to adsorption and/or DNA hydrolysis, denaturation, and depurination at alkaline to acidic conditions (Gates, 2009). These interactions are further exacerbated in low-biomass environments and by nanopore sequencing, which presently requires substantial (1 μg or 400 ng of high molecular weight) input DNA not likely to be acquired from recalcitrant soils (without amplification) due to limitations related to sequencing efficiency and nanopore longevity (Mojarro *et al.,* 2018). Roughly five in a million nucleobases are sequenced (R9.4 flowcells and chemistry) while nanopores are expended before detection of sub-nanogram input DNA (Mojarro *et al.,* 2017a, 2018) without library preparation modifications.

Extensive literature exists on methods used to mitigate soil-DNA interactions and yield quantifiable DNA from various soil species (*e.g.,* Takada-Hoshino and Matsumoto, 2004; Barton *et al.,* 2006; Henneberger *et al.,* 2006; Herrera and Cockell, 2007; Direito *et al.,* 2012; Lever *et al.,* 2015). Competitive binders or blocking agents such as RNA, random hexamer primers, sodium pyrophosphate, and skim milk (*e.g.,* Takada-Hoshino and Matsumoto, 2004) are applied to inhibit mineral adsorption sites while dialysis, desalting, or chelation (*e.g.,* Barton *et al.,* 2006) is employed to precipitate or flush soluble metals and salts from soils. Our previous work from Mojarro *et al.* (2017b) focused on adapting the standard Purelyse bacterial gDNA extraction kit to isolate DNA from Mars analog soils doped with tough-to-lyse spores of *Bacillus subtilis*. In that study, we concluded that with soil-specific mitigation strategies (*e.g.,* desalting and competitive binders), we may be able to achieve adequate DNA extraction yields that are of sufficient purity for downstream sequencing.

In this study, we now focus on validating the modified Purelyse kit with low-biomass Mars analog soils containing cell densities observed in the driest regions of the Atacama Desert (Navarro-Gonzalez *et al.,* 2003) and McMurdo Dry Valleys (Goordial *et al.,* 2016). We perform baseline and modified DNA extractions from 50 mg of Mars analog soil containing 10^4^ spores of *B. subtilis* and vegetative *Escherichia coli* cells, respectively. These experiments stress the importance of the mitigation strategies used to reduce detrimental soil-DNA interactions (*e.g.,* adsorption to mineral surfaces, DNA destruction) and conclude in the development of a “near-universal” extraction protocol for low-biomass Mars analog soils. As in our previous experiments (Carr *et al.,* 2016, 2017), we require DNA extraction yields of at least 5% in order to achieve a minimum sensitivity target of 1 ppb for life detection (*i.e.,* 2 pg of DNA from 10^4^ cells in 50 mg of soil).

Lastly, we investigate low-input nanopore sequencing by experimenting with 2 pg of purified *B. subtilis* spore DNA equivalent to a 5% DNA yield from 10^4^ spores. For many environmental samples, the total extractable DNA is far below the current input requirements of nanopore sequencing, preventing sample-to-sequence metagenomics from low-biomass or recalcitrant samples. On Mars, whole genome amplification could result in biasing microbial population results (Sabina and Leamon, 2015) while targeted amplicon sequencing such as 16S rRNA would assume a shared ancestry between Mars and Earth and could reduce taxonomic resolution (Poretsky *et al.,* 2014). The absence of amplification is especially important in the case where nonstandard bases have been incorporated into the martian genome, as primers may not function or amplification could theoretically mask an alien signal due to promiscuous base-pairing resulting in an ATGC sequence (Pezo *et al.,* 2014). Here we address these problems by employing carrier sequencing, a method to sequence low-input DNA by preparing the target DNA with a genomic carrier to achieve ideal library preparation and sequencing stoichiometry without amplification (Raley *et al.,* 2014; Mojarro *et al.,* 2018). We then use CarrierSeq (https://github.com/amojarro/carrierseq, Mojarro *et al.,* 2018), a sequence analysis script used to identify low-input target (unknown) reads from the genomic carrier, and analyze the results in the context of life detection.

## 2. Materials and Methods

### 2.1. Mars analog soils

A total of six Mars analog soils and one lunar basalt analog (Orbitec, JSC-1A) (McKay *et al.,* 1993) were utilized to develop our extraction protocols. Five Mars analog soils were produced in accordance with *in situ* mineralogical and geochemical measurements collected by rover and lander missions as described by Schuerger *et al.* (2012, 2017). These soils represent various potentially habitable paleoenvironments of astrobiological significance, for instance, ancient hydrothermal spring salt deposits in Gusev Crater (Ming *et al.,* 2006; Ruff and Farmer, 2016). The five soils represent (1) the highly oxidized global aeolian dust (Bell *et al.,* 2000; Ming *et al.,* 2008a), (2) salt-rich (Burroughs subclass) soils of Gusev Crater (Ming *et al.,* 2006; Morris *et al.,* 2008), (3) jarosite-containing acidic soils (Paso Robles class) of Meridiani Planum (Klingelhofer, 2004; Morris *et al.,* 2006; Ming *et al.,* 2008a), (4) carbonate-rich alkaline Viking lander soils (Clark *et al.,* 1982; Wänke *et al.,* 2001), and (5) perchlorate-rich Phoenix lander soils (Ming *et al.,* 2008b). In addition, we include a commercially available (6) aeolian spectral analog (Orbitec, JSC-Mars-1A) (Allen *et al.,* 1998). We henceforth refer to these Mars analog soils as aeolian, salt, acid, alkaline, perchlorate, and JSC, respectively. The lunar analog represents our unaltered soil control and is referred to as basalt. Furthermore, the basalt sample closely resembles the reduced martian subsurface (Taylor, 2013; Grotzinger *et al.,* 2014), which could theoretically harbor a deep modern biosphere (Jones *et al.,* 2011). All soils were heat-sterilized at 130°C for 48 h prior to any experimentation in a Fisher Science Isotemp 282A vacuum oven, cooled overnight, and stored in air-tight borosilicate bottles (Corning, 1395-100) at room temperature (Schuerger *et al.,* 2012). Details concerning the exact methods used to synthesize these soils can be found in the works of Schuerger *et al.* (2012) and Mojarro *et al.* (2017b).

### 2.2.* Bacillus subtilis *spores

Spore suspensions of *Bacillus subtilis* (ATCC 6633) similar to those used in clean-room sterilization effectiveness (Friedline *et al.,* 2015) and bacteriostasis testing under Mars-like conditions (Kerney and Schuerger, 2011; Schuerger *et al.,* 2017, 2003) were acquired from Crosstex (Part# SBS-08) to represent a worst-case DNA extraction scenario of a tough-to-lyse organism. Spore suspensions were DNAse-treated (New England Biolabs, M0303L) in order to remove any extracellular DNA and counted using a viable spore assay on lysogeny broth agar plates (Mojarro *et al.,* 2017b). We assume a single genome copy per DNAse-treated spore of *B. subtilis* for calculating DNA yield (DNA_out_/DNA_in_).

### 2.3. Vegetative *Escherichia coli* cells

Vegetative *Escherichia coli* (OP50) cell cultures were grown overnight in flasks containing 200 mL of lysogeny broth medium inside an Innova 44 incubator at 37°C and 200 rpm. After 12 h, we measured optical density (OD600) on a DeNovix DS-11+ spectrophotometer, pelleted, and resuspended culture aliquots in phosphate-buffered saline solution (Thermo Fisher, 10010023). An estimated 1000 *E. coli* cells then underwent colony droplet digital polymerase chain reaction (ddPCR) with single copy *metG* (Wang and Wood, 2011) primers for absolute genome quantitation. In contrast to spores that contain a single genome copy, copy-number variation may exist in vegetative cells depending on growth stage (Skarstad *et al.,* 1986; Pecoraro *et al.,* 2011). Therefore, ddPCR results allow us to correct for genome copy-number variation (*e.g.,* 2000 genome copies / 1000 cells) and calculate DNA yield similar to *B. subtilis* (DNA_out_/DNA_in_).

### 2.4. DNA yield quantitation

Absolute DNA yield quantitation is possible through ddPCR paired with single copy primers. In contrast to traditional PCR, ddPCR fractionates one reaction into ∼20,000 water-oil emulsion nanodroplets each capable of discrete amplification. When a sample is adequately dilute, Poisson statistics can precisely determine genome copy numbers independent of a standard curve (Brunetto *et al.,* 2014; Yang *et al.,* 2014). Paired with single copy primers and the presence of a DNA-specific fluorescent dye (EvaGreen), the results of a ddPCR reaction are either relatively weakly fluorescent droplets containing primer dimer or relatively highly fluorescent droplets containing an amplified product. We then equate one highly fluorescent droplet to one genome copy. DNA extracted from *B. subtilis* spores was amplified with *spaC* primers (forward: TGA GGA AGG ATG GGA CGA CA, reverse: AAC AGA TTG CTG CCA GTC CA) (Hu *et al.,* 2004) while DNA extracted from vegetative *E. coli* cells was amplified with *metG* primers (forward: GGT GGA AGC CTC TAA AGA AGA AG, reverse: AGC AGT TTG TCA GAA CCT TCA AC) (Wang and Wood, 2011). Two ddPCR reactions per DNA extraction (one per elution) were performed and quantified on a BioRad QX200 ddPCR system. A total of 5 μL from each extraction elution was prepared in a 20 μL final reaction volume containing 3 μL of molecular-grade water (Thermo Fisher, 10977023), 10 μL of QX200 EvaGreen SuperMix (BioRad, 186-4033), 1 μL of 3.3 μM forward primer, and 1 μL of 3.3 μM reverse primer. All ddPCR reactions were prepared by an Andrew Alliance liquid handling robot within an AirClean 600 PCR Workstation while nanodroplets were generated using the automated QX200 AutoDG system. Thermocycling conditions were as follows: (1) 95°C for 5 min, (2) 95°C for 30 s, (3) 60°C for 1 min, (4) repeat steps 2–3 40 times, (5) 4°C for 5 min, (6) 90°C for 5 min, (7) hold at 4°C until ready to measure.

### 2.5. Purelyse standard extraction protocol

The Purelyse bacterial gDNA extraction kit is a miniature (dime-sized) and battery-powered bead-beating lysis device capable of solid-phase nucleic acid extraction without the need for centrifugation. Purelyse kits work by shearing cells open at 30,000 rpm in the presence of a supplied low-pH (∼3.5 pH) binding buffer that promotes the binding of negatively charged polymers to the surface of specialty oxide ceramic microbeads (∼100 μm). The microbeads are then washed with a lower-concentration binding buffer, and DNA is eluted in a low-salt and high-pH (∼8.0 pH) elution buffer. The standard extraction protocol for cell cultures dictates: (1) 2 min lysis with 1× binding buffer solution (100 μL of 8× binding buffer and 700 μL of molecular-grade water), (2) 45 s wash with 3 mL of 0.25× binding buffer wash solution (∼100 μL of 8× binding buffer and 2.9 mL of molecular-grade water), (3) two 1 min elutions with 200 μL of 1× elution buffer all at 6V (equivalent to 4 AAA batteries).

### 2.6. Baseline extractions of spore and vegetative DNA from water

Two dilution series containing 10^8^, 10^6^, and 10^4^ spores of *B. subtilis* and equivalent vegetative *E. coli* cells in water were processed using the standard extraction protocol. The *E. coli* dilution series originated from a single culture prepared in phosphate-buffered saline solution, and DNA yield was corrected for genome copy-number variation using ddPCR.

### 2.7. Unmodified extractions of spore and vegetative DNA from Mars analog soils

An estimated 1.6 × 10^4^ spores (about 70 pg of DNA) of *B. subtilis* were deposited on 50 mg of Mars analog soil and processed following the standard extraction protocol. For vegetative *E. coli* extractions, we utilized OD600 measurements to deposit approximately 1.6 × 10^4^ cells on 50 mg of Mars analog soil or equivalent to spore concentrations. However, as *E. coli* genome copy number may vary (Skarstad *et al.,* 1986; Pecoraro *et al.,* 2011), each *E. coli* extraction from a Mars analog included accompanying colony ddPCR results used for correcting genome copy-number variation.

### 2.8. Modified extractions of spore and vegetative DNA from Mars analog soils

To parallel the unmodified extractions, an estimated 1.6 × 10^4^ spores of *B. subtilis* and vegetative cells of *E. coli* were deposited on 50 mg of Mars analog soil, respectively. All samples were processed by using the “near-universal” modified extraction protocol: (1) Suspend sample in 800 μL of 8 × binding buffer and vortex gently for 30 s, (2) desalt in a single 100K Amincon Ultra column (Z740183), (3) resuspend the sample in 400 μL of molecular-grade water and 400 μL of 8 × binding buffer, (4) add 4–6 μg of random hexamer primers (Promega, C1181) and vortex gently for 30 s, (5) lyse cells and bind DNA with Purelyse at 6.5 V for 2 min, (6) wash with 2.5 mL of 1 × binding buffer at 1.5 V for 1 min, (7) elute DNA with 200 μL of heated elution buffer to 70°C for 1 min, (8) repeat step 7 for second elution. Basalt and alkaline samples contained 4 μg of random hexamer primers as we observed a decrease in DNA yield with increasing amounts. We believe this is most likely due to hexamer-induced competitive binding since basalt and alkaline samples have a lower DNA binding affinity (Mojarro *et al.,* 2017b). Meanwhile JSC, acid, salt, aeolian, and perchlorate samples required 6 μg.

### 2.9. Nanopore sequencing

The Oxford Nanopore Technologies MinION sequencer and flowcell perform single-strand DNA sequencing through monitoring changes in an ionic current produced by the translocation of k-mers through a nano-sized pore (Lu *et al.,* 2016). Nanopore sequencing encompasses library preparation, sequencing, and basecalling. First, library preparation is the process by which genomic DNA is converted into a readable format for the sequencer. This includes adding tethers and a motor protein onto double-stranded DNA which guide and regulate the translocation rate (R9.4, 450 bp/s) through a nanopore. Second, the prepared library is loaded onto the flowcell, and sequencing is initiated. Up to 512 sequencing channels (pores) can be monitored, and raw current (translocation) events are recorded. Lastly, the raw events are then basecalled into corresponding nucleobase assignments or oligomer sequences.

### 2.10. *Bacillus subtilis* spore DNA extraction for low-input nanopore sequencing

To simulate an ideal sample-to-sequence scenario, an estimated 2.0 × 10^8^ spores of *B. subtilis* in water were processed by using the standard extraction protocol. DNA yield was then quantified using a double-stranded, DNA-specific fluorometric assay (Invitrogen, Qubit dsDNA HS Assay Kit, Q32854) and a Qubit 2.0 fluorometer (Invitrogen, Q32866, limit of detection of 0.1 ng/mL, dsDNA). Once the DNA concentration was known, an aliquot of the purified spore DNA was diluted in molecular-grade water to 2 pg/μL and subsequently verified with ddPCR prior to library preparation.

### 2.11. Low-input sequencing library preparation

The sequencing library was prepared following a modified one-direction (1D) Lambda control experiment protocol (Oxford Nanopore Technologies, SQK-LSK108). The ligation-based sequencing kit advises shearing 1 μg of Lambda genomic DNA to 8 kb fragments and spiking the sheared gDNA with a 3.6 kb positive control (Lambda genome 3'-end amplicon) in order to distinguish library preparation from sequencing failures. However, after validating our library preparation proficiency, we substituted 2 pg of *B. subtilis* spore DNA purified with Purelyse in lieu of the 3.6 kb positive control and replicated the Lambda control protocol without additional modifications. This substitution results in a low-input carrier library with ideal stoichiometry (Mojarro *et al.,* 2018).

### 2.12. Sequencing and basecalling

The low-input carrier library was sequenced by a MinION Mk 1B sequencer and R9.4 spot-on flowcell for 48 h on an Apple iMac desktop computer running MinKNOW 1.5.18. The resulting raw nanopore reads were basecalled with Oxford Nanopore Technologies' Albacore 1.10 offline basecaller.

### 2.13. Sequence analysis

All reads located in the Albacore workstation folder were compiled into a single fastq file and mapped directly to the *B. subtilis* reference genome using bwa (Li, 2013). In the context of an unknown sample, all reads were then processed with CarrierSeq (Mojarro *et al.,* 2018), a sequence analysis script for carrier sequencing. CarrierSeq works by identifying all reads not belonging to the carrier (Lambda), applies quality-control filters, and implements a Poisson test to identify likely nanopore sequencing artifacts known as high-quality noise reads (HQNRs), which presumably originate from malfunctioning nanopores. The final subset or “target reads” should therefore only contain *B. subtilis* and likely contamination (Mojarro *et al.,* 2018).

## 3. Results

### 3.1. Baseline extractions of spore and vegetative cell DNA from water

**FIG. 1.**
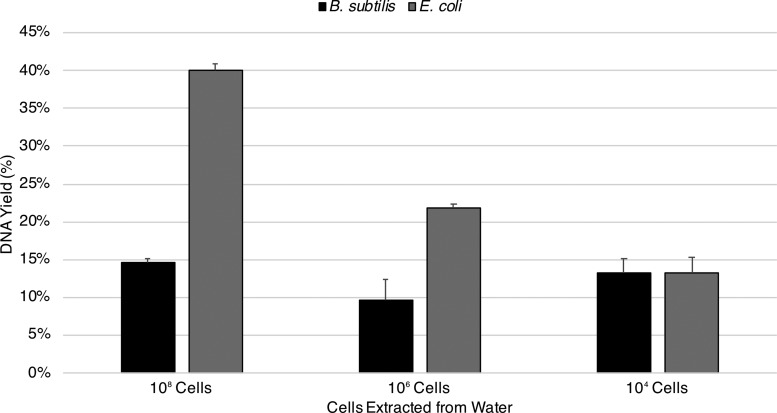
Baseline extractions of spore and vegetative cell DNA from water. Two dilution series containing 10^8^, 10^6^, and 10^4^ spores of *B. subtilis* and equivalent vegetative *E. coli* cells were processed using the standard extraction protocol. Water extractions of spore DNA remained consistent throughout the dilution series. In contrast, extractions of vegetative cell DNA decreased with cell concentrations (standard error shown, *n* = 3).

Water extractions of spore DNA remained consistent throughout the dilution series. At 10^8^, 10^6^, and 10^4^ spores, yields were 14.7%, 9.7%, and 13.3% (Fig. 1). In contrast, extractions of vegetative cell DNA decreased with cell concentrations. At 10^8^, 10^6^, and 10^4^ vegetative cells, yields were 40%, 21.8%, and 13.2% (Fig. 1).

### 3.2. Unmodified extractions of spore and vegetative cell DNA from Mars analog soils

**FIG. 2.**
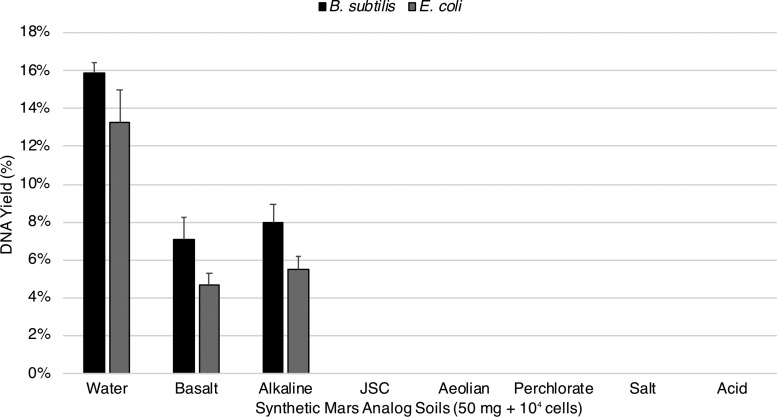
Unmodified extractions of spore and vegetative cell DNA from Mars analog soils. An estimated 1.6 × 10^4^ spores (about 70 pg of DNA) of *B. subtilis* and equivalent vegetative cells of *E. coli* were deposited on 50 mg of Mars analog soil and processed following the standard extraction protocol. No detectable DNA was measured from any Mars analog soil extraction containing either spores or vegetative cells with the exception of the basalt and alkaline soils (standard error shown, *n* = 3).

No detectable DNA was measured from any Mars analog soil extraction containing either spores or vegetative cells with the exception of the basalt and alkaline soils. Unmodified spore DNA extractions yielded 7.1% for basalt and 8% for alkaline while vegetative DNA extractions yielded 4.7% for basalt and 5.5% for alkaline (Fig. 2).

### 3.3. Modified extractions of spore and vegetative cell DNA from Mars analog soils

**FIG. 3.**
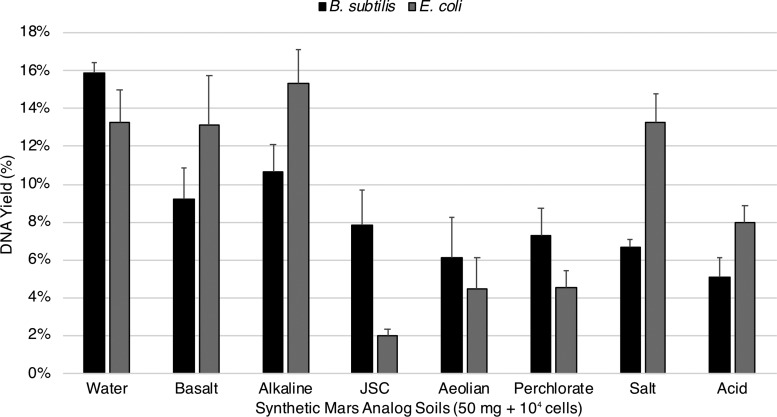
Modified extractions of spore and vegetative DNA from Mars analog soils. All DNA yields from 10^4^ vegetative cells and spores in 50 mg of Mars analog soils were increased with the “near-universal” extraction protocol. Our results indicate that a combination of desalting and completive binders is an adequate strategy for achieving our extraction goals (standard error shown, *n* = 3).

All modified Mars analog soil extractions of spore DNA achieved our 5% requirement by using the “near-universal” protocol. DNA yields were modestly increased for alkaline and basalt samples while aeolian, salt, acid, perchlorate, and JSC samples increased significantly from undetectable amounts. The spore results were basalt 9.2%, alkaline 10.7%, aeolian 6.1%, salt 6.7%, acid 5.1%, perchlorate 7.3%, JSC 7.8% (Fig. 3). In contrast, several vegetative cell DNA extractions, for example, JSC, aeolian, and perchlorate, failed to achieve 5% DNA yield. Similar to the spore extractions, yields for alkaline and basalt samples received a moderate increase while the remaining samples increased from undetectable amounts. The vegetative cell results were basalt 13.1%, alkaline 15.3%, aeolian 4.4%, salt 13.3%, acid 8.0%, perchlorate 4.5%, JSC 2.0% (Fig. 3).

### 3.4. Nanopore sequencing

From the resulting 48 h of sequencing of purified *B. subtilis* spore DNA in the presence of Lambda, we detected a total of 8.7 gigabases or 1.3 million reads. Exactly 1,260,661 reads mapped to Lambda while 5 reads mapped to *B. subtilis*. Given the sequencing library's Lambda to *B. subtilis* mass ratio, we expected approximately 2 *B. subtilis* reads per 1,000,000 Lambda reads. However, we detected ∼4 *B. subtilis* reads per 1,000,000 Lambda reads. Assuming Lambda is the only known sequence, the CarrierSeq analysis (*Q* = 9, *p* = 0.05, default values) isolated 29 “target reads” (Table 1) that were identified using the NCBI blastn algorithm: 5 *B. subtilis* reads, 13 *Homo sapiens* reads, 1 *Klebsiella pneumoniae* read, 1 human mastadenovirus read, 1 human DNA sequence (not able to be resolved to *Homo sapiens*), 1 *Streptococcus* sp. read, 1 *Staphylococcus epidermis* read, and 6 unidentified HQNRs (Table 2). The basecalled and unanalyzed sequencing fastq files can be downloaded from FigShare at https://doi.org/10.6084/m9.figshare.5471824.v2, and the raw fast5 files can be downloaded from the NCBI SRA database at https://www.ncbi.nlm.nih.gov/biosample/9534236.

**Table 1.** Low-Input Carrier Sequencing Metrics

	*Number of reads*		*Min length*		*Max length*		*Median length*		*Total bases*	
All reads (Lambda + *B. subtilis* + Contamination + HQNRs)	1,303,007	reads	16	bases	501,249	bases	6,493	bases	8,698,026,598	bases
Target reads (*B. subtilis* + Contamination + HQNRs)	29	reads	267	bases	1,559	bases	595	bases	19,981	bases
*B. subtilis* reads	5	reads	848	bases	1,559	bases	967	bases	5,270	bases
Contamination reads HQNRs	6	reads	267	bases	865	bases	453	bases	2,933	bases

**Table 2.** Target Reads

*Organism*	*Read length*		*Quality score*	*DUST score*	*Query cover*		*E value*	*Identity*		*Read ID*
*Bacillus subtilis.1*	848	bases	11.7	1.81	94	%	0	89	%	@channel_69_7447cdae-d9cc-4ce8-a7f5-b0602b74f485_template
*Bacillus subtilis.2*	967	bases	11.7	1.79	96	%	0	89	%	@channel_200_5dbffb79-af02-47d8-9b80-4bladaab20e8_template
*Bacillus subtilis.3*	1559	bases	11.4	1.85	96	%	0	86	%	@channel_217_4e9b9a3a-4d3f-4e85-9287-1607085867c5_template
*Bacillus subtilis.4*	872	bases	10.0	1.70	94	%	0	83	%	@channel_381_2ed7f3f3-ad20-4529-a63e-3af8639d1769_template
*Bacillus subtilis.5*	1024	bases	11.2	1.77	96	%	0	86	%	@channel_500_337c80cf-a4b9-403d-b0b3-71c443339b85_template
*Streptococcus* sp.	1556	bases	11.1	2.08	94	%	0	84	%	@channel_149_9b525218-4812-46a9-b8ca-dd8e0cc326b6_template
*Staphylococcus epidermis*	561	bases	9.39	2.09	92	%	8.E-129	83	%	@channel_376_9f4bcd65-7083-44ca-8ee1-b20c2b22df2e_template
*Klebsiella pneumoniae*	527	bases	11.6	1.59	91	%	0	91	%	@channel_491_2e67e9b2-8a67-41da-b4ca-d3ba18248350_template
Human mastadenovirus	595	bases	9.45	1.93	90	%	3.E-148	85	%	@channel_434_cc74d4a9-b62f-4274-86d0-7d95370b6aba_template
Human DNA sequence	627	bases	9.47	1.95	57	%	7.E-100	85	%	@channel_408_44516fbc-2f75-46e6-870e-953c50ac6112_template
*Homo sapiens.1*	681	bases	11.4	2.08	93	%	0	90	%	@channel_13_7738ebc8-3fdc-4cf2-9854-9b18b81ec924_template
*Homo sapiens.2*	496	bases	10.2	3.12	91	%	1.E-91	81	%	@channel_182_5ed727df-dd19-481e-9820-f77f5c20d1e9_template
*Homo sapiens.3*	416	bases	12.1	1.84	92	%	1.E-127	88	%	@channel_227_f3bf082a-827e-4557-a9db-20f662dfce22_template
*Homo sapiens.4*	989	bases	10.9	2.02	95	%	0	86	%	@channel_86_e52fe73a-e4e7-45d5-9a72-71c473e18cb4_template
*Homo sapiens.5*	434	bases	11.9	1.89	86	%	2.E-135	90	%	@channel_98_ac4b4212-6fd1-49cd-9042-38fb46a943ce_template
*Homo sapiens.6*	638	bases	11.7	1.89	90	%	0	89	%	@channel_101_350b8de1-2288-4109-8b4a-a2210c0dd22e_template
*Homo sapiens.7*	569	bases	11.9	2.29	92	%	6.E-180	88	%	@channel_139_239119b0-d638-4d8b-a2f2-f6ffb8aa0003_template
*Homo sapiens.8*	933	bases	9.35	2.10	93	%	0	83	%	@channel_368_2edf50ed-1c62-42f0-ba32-0c05229ab9a2_template
*Homo sapiens.9*	554	bases	10.1	1.99	91	%	2.E-150	86	%	@channel_375_97c139b2-fc1a-4a9f-96f8-4ce66247dbe2_template
*Homo sapiens.10*	661	bases	10.1	2.38	93	%	2.E-164	84	%	@channel_447_472aece4-a8c2-4090-bd1a-0a0e6e35eec1_template
*Homo sapiens.11*	654	bases	11.6	2.44	95	%	0	89	%	@channel_290_d2806fbe-0e0b-43f6-aa24-027e1f481630_template
*Homo sapiens.12*	445	bases	9.91	1.76	96	%	1.E-132	87	%	@channel_300_a9532014-5520-48fd-9d3a-6f8f52c45eeb_template
*Homo sapiens.13*	442	bases	9.65	2.00	91	%	9.E-59	78	%	@channel_318_b634ac5d-4a8d-401d-9dff-1314985e8fea_template
HQNR.1	581	bases	12.7	2.44	—		—	—		@channel_334_1b63719e-2745-4eb8-afdf-7c5c19d94ec4_template
HQNR.2	422	bases	9.18	5.99	—		—	—		@channel_143_a198d662-4306-42a5-bd53-caf57c7744fc_template
HQNR.3	267	bases	9.22	2.21	—		—	—		@channel_54_103c006a-34c3-45ec-a521-ccbf7b461e5f_template
HQNR.4	865	bases	9.16	1.85	—		—	—		@channel_156_54ea11b1-5323-4805-a7b8-53f0840d40de_template
HQNR.5	484	bases	11.7	1.94	—		—	—		@channel_166_f5014250-90d6-4977-9307-3782e7e47a0d_template
HQNR.6	314	bases	9.42	1.98	—		—	—		@channel_241_19270801-958b-418b-a5fc-7f8b02179efa_template

## 4. Discussion

### 4.1. Extraction of DNA from synthetic Mars analog soils

The extractions from spore DNA in water and soils at 10^4^ spores yielded similar results to our prior experiments at 10^8^ spores used to develop the initial modified protocol (Mojarro *et al.,* 2017b). We previously hypothesized that low DNA yields from *B. subtilis* were generally due to small acid-soluble proteins (SASPs) which bind to the DNA phosphate backbone and furnish protection from heat, salts, desiccation, and UV radiation in the spore state (Moeller *et al.,* 2009, 2012). In short, SASPs could inhibit interactions between the phosphate backbone and the Purelyse oxide ceramic microbeads, which would ideally attract negatively charged polymers (*i.e.,* DNA). In the work of Mojarro *et al.* (2017b), we seemingly validated this hypothesis by increasing spore DNA yields in water from 15% to 43% using a proof-of-concept protein separation binding buffer/phenol cocktail. We therefore intuitively expected the DNA extraction yields from vegetative *E. coli* cells, which presumably do not contain SASPs, to be higher than *B. subtilis* spores. Extractions of spore DNA from our water control consistently yielded ∼15% at all cell concentrations while extractions of vegetative DNA began with 40% at 10^8^ cells and decreased to 13.3% at 10^4^ (Fig. 1). These extractions share the exact parameters across cell lysis and DNA elution voltage, time, buffer volumes, and approximate cell concentrations with the exception of cells being either spores or vegetative. We are presently unable to offer a definitive explanation for the decrease in vegetative DNA with decreasing cell concentration and the apparent convergence of DNA yields at 10^4^ cells (Fig. 1). Perhaps (1) cell concentration may affect DNA yield, (2) binding of nucleic acids to the Purelyse oxide ceramic microbeads is proportional to the concentration of lysed DNA, (3) vegetative DNA is more prone to destruction (*e.g.,* hydrolysis, etc.) in solution without SASPs, or (4) vegetative DNA has a stronger affinity toward the Purelyse oxide ceramic microbeads and/or soil. Further work is ongoing in order to investigate this inconsistency between spore and vegetative DNA yields.

In regard to the Mars analog soil extractions, prior work from Mojarro *et al.* (2017b) had suggested that both alkaline and basalt soils would exert mild soil-DNA interactions due to adsorption effects by silicates (Melzak *et al.,* 1996; Trevors, 1996; Zhou *et al.,* 1996) while highly oxidized iron sulfate-containing Mars analog soils would undoubtedly destroy most DNA in an unmodified extraction (Fig. 2). Nevertheless, once disruptive metals/cations (*e.g.,* Fe^3+,2+^, Ca^2+^) were flushed and mineral adsorption sites were coated with competitive binders, DNA yields of at least 5% were achieved in all soils containing spores of *B. subtilis* (Fig. 3). Extractions from vegetative *E. coli* cells were only capable of achieving a 5% yield within the standard error with the exception of JSC, which yielded 2.0% (Fig. 3). These results indicate that perhaps DNA bound by SASPs has an increased resistance to destruction in the soil/binding buffer slurry immediately after cell lysis. If so, it would appear that DNA is more susceptible to damage from free radicals in Mars analog soils containing Fe^3+,2+^ species (JSC, aeolian, perchlorate) than from hydrolysis, depurination, and denaturation in acidic to alkaline soils (acid, salt, alkaline) (Fig. 3).

### 4.2. Ancient DNA on Mars

The relatively stable cryosphere on Mars since the Late Noachian period (Head and Marchant, 2014) makes it the ideal location for ancient DNA preservation. On Earth, the estimated half-life of DNA at -25°C is roughly on the order of 10^7^ years (Millar and Lambert, 2013). However, local and global natural climate variability due to orbital cycles (Imbrie *et al.,* 1992) and tectonics (Raymo and Ruddiman, 1992) limit the extent of cold environments favorable toward ancient DNA preservation on geologic timescales. Today, the average temperature at Gale Crater is -48°C (Haberle *et al.,* 2014) while similar conditions may have persisted since the Amazonian period (Head and Marchant, 2014), theoretically permitting the preservation of DNA beyond 10 million years. However, the likelihood of ancient DNA on the surface of Mars greatly decreases once we consider the effect of UV, cosmic ray exposure, and radioactive decay on nucleic acids (Kminek *et al.,* 2003; Kminek and Bada, 2006; Hassler *et al.,* 2014). Ancient DNA is prone to damage (*e.g.,* hydrolysis, depurination) and fragmentation (Dabney *et al.,* 2013; Millar and Lambert, 2013) once it is no longer actively repaired in a biologic system. In the unlikely case that nucleic acids are preserved and accessible at the martian surface, we then risk the possibility of destroying them once soils are hydrated during an extraction if they are not protected within cells (Gates, 2009; Mojarro *et al.,* 2017b). The primary target for SETG is therefore extant or recently dead cells encapsulating nucleic acids that may survive the sample prep processes and yield long strands useful for taxonomic identification.

### 4.3. Low-input nanopore sequencing and contamination detection

A great advantage of nanopore sequencing is the ability to produce long reads capable of taxonomic identification (Greninger *et al.,* 2015; Quick *et al.,* 2015; Brown *et al.,* 2017; Goordial *et al.,* 2017). This has allowed us to directly map all basecalled reads to the reference genome and identify the five belonging to *B. subtilis* without further processing. However, in a true life-detection or unknown scenario, there must be a way to filter carrier (Lambda), low-quality, and low-complexity reads in order to identify those belonging to the unknown organisms. As mentioned earlier, we used CarrierSeq, which was developed in order to analyze carrier sequencing runs and identify the unknown or “target reads” in this context. This analysis identified 29 reads which did not map to Lambda, were not considered low quality (*Q* < 9, Albacore passing was *Q* > 6 at the time of analysis, currently *Q* > 7) or low complexity (Morgulis *et al.,* 2006), and did not originate from overly active “bad pores” that are known to produce spurious reads which we refer to as high-quality noise reads (HQNRs) in the work of Mojarro *et al.* (2018). These include the 5 *B. subtilis* reads detected by direct mapping, 6 HQNRs that originated from “good pores,” and most interestingly, 18 *Homo sapiens* and human microflora contamination reads (Table 2). Although we cannot recollect an exact point of contamination, this work was conducted in an open lab, with nonsterile nitrile gloves, and in an open bench without any nonstandard precautions (*e.g.,* routine sporicidal/bleaching was conducted between sets of extractions).

**FIG. 4.**
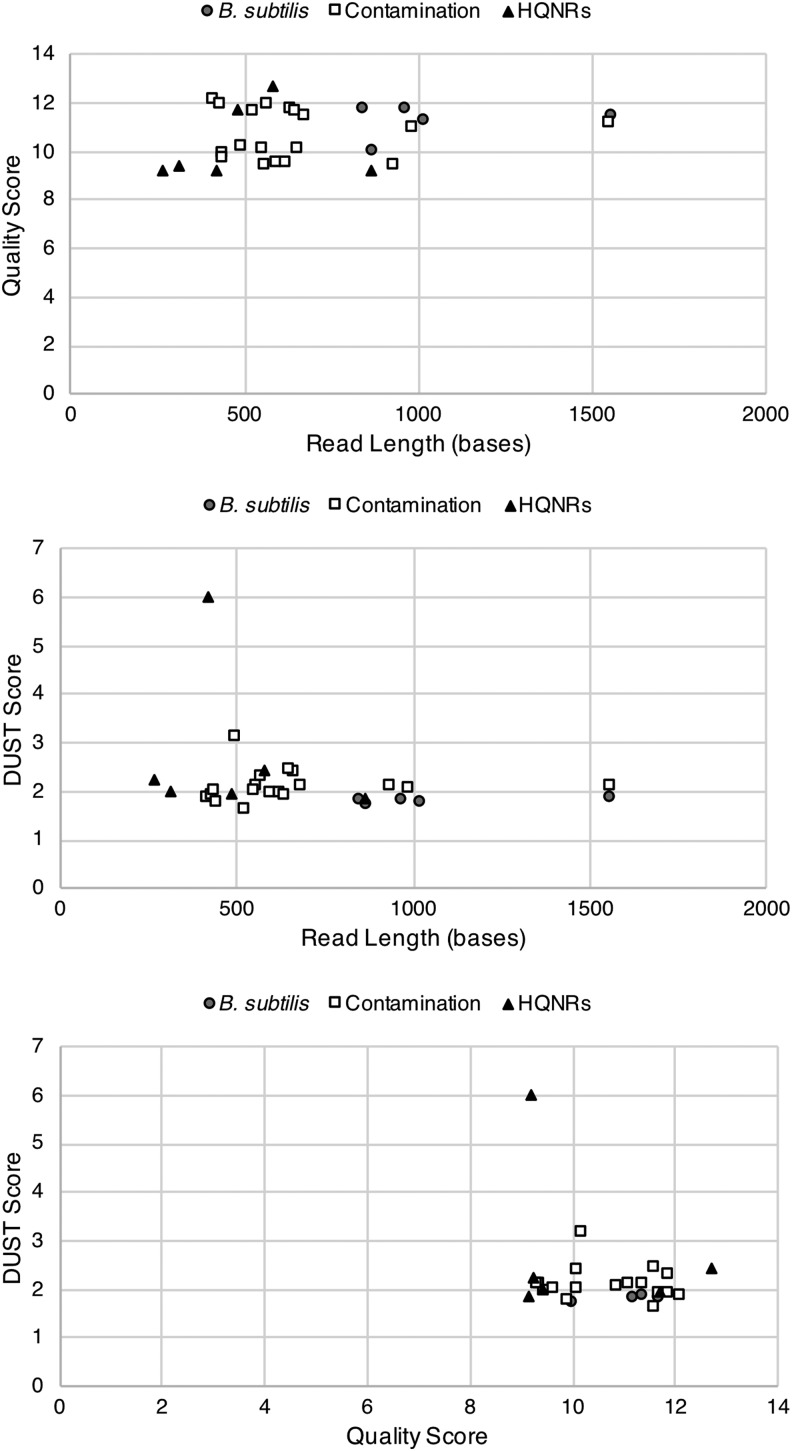
Cross-plots of target reads identified by CarrierSeq displaying read length, quality score, and DUST score. There does not appear to be any separation of HQNRs and *B. subtilis* or contamination reads. We are presently only able to identify HQNRs through k-mer matching or via the NCBI blastn algorithm.

**FIG. 5.**
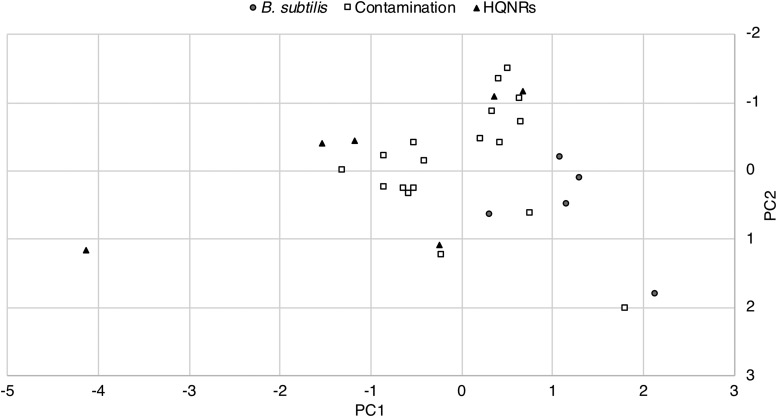
Principal component analysis of the CarrierSeq target reads (*n* = 29). There does not appear to be any separability based on read length, quality score, and DUST score parameters.

The close abundance of contamination to *B. subtilis* reads possibly suggests comparable low levels of contamination (2 pg of *B. subtilis*), although there is no certain strategy to ascertain the true initial contamination quantities. We instead see this as an opportunity to highlight the effectiveness of nanopore sequencing to discriminate between true detection and contamination, particularly in the context of planetary protection where carrier sequencing could be employed as an effective method of detecting low levels of microbial contamination on the surface of spacecrafts (Rummel and Conley, 2018). The presence of HQNRs, however, complicates the certainty between a true unknown read and noise. Mojarro *et al.* (2018) proposed that HQNRs could possibly be the result of pore blockages from macromolecules (*e.g.,* proteins) that may conceivably produce false signals. To date, we have not produced a fully satisfactory approach to identify and discard such reads. Identifying HQNRs through k-mer matching (Menzel *et al.,* 2016) and via the NCBI blastn algorithm is presently the best approach, while plotting DUST (Morgulis *et al.,* 2006), quality (Phred) score, and read length and statistical tests do not reveal any obvious relationships (Figs. 4 and 5). Recent work by Pontefract *et al.* (2018) on the failure modes of nanopore sequencing has suggested that improvements to basecalling algorithms, library preparation, and sequencing chemistries have suppressed the occurrence of these spurious reads. However, as Oxford Nanopore Technologies matures its sequencing technology, more robust testing under simulated low-input and unknown sample conditions is required in order to confidently identify HQNRs (if they are still an issue) where these artifactual reads may complicate the identification of a new organism or, specifically, extraterrestrial life.

### 4.4. Detecting nonstandard bases

Raw nanopore sequencing data represent the translocation of unique k-mers, or nucleotide sequences of k bases, through a critical pore region which produces an associated ionic current signal. For simplicity, let us imagine a sliding window that is 3 bases wide (a 3-mer) moving one base at a time over a single-stranded DNA of sequence AATCG. As the window slides, we should observe AAT, ATC, and TCG each producing a unique signal. A moving 3-mer window over a DNA strand containing the four standard nucleobases (ATCG) could encounter up to 64 distinctive combinations. Therefore, a DNA strand containing a theoretical fifth nonstandard base would have 125 possible combinations, and so on. Raw data is then basecalled by applying algorithms such as Hidden Markov Models (Simpson *et al.,* 2016) or Recurrent Neural Networks (Boža *et al.,* 2017) that translate the collection of k-mers into consensus nucleic acid sequences. In previous work, we have demonstrated the capacity for these algorithms to detect nonstandard bases by sequencing poly(deoxyinosinic-deoxycytidylic) acid, a synthetic DNA polymer composed of alternating deoxy-inosine (I) and deoxy-cytosine (C) bases (Carr *et al.,* 2017). This proof of concept is of particular interest since the nonstandard inosine nucleotide contains hypoxanthine, a nucleobase that has been identified within meteorites (Martins *et al.,* 2008). Meanwhile, work by others has also demonstrated the ability to detect base modification such as methylation (Simpson *et al.,* 2016) using novel basecalling algorithms.

## 5. Conclusions

Life on Mars, if it exists or existed in the not-so-distant past, may potentially be detected via *in situ* nucleic acid extraction and nanopore sequencing. In this study, we have validated methodologies that enable the extraction of nucleic acids from low-biomass and recalcitrant analog soils ranging from a variety of surface environments (*e.g.,* ancient hydrothermal spring deposits) to the subsurface of Mars. Our results indicate that a combination of desalting and competitive binding is a viable approach for achieving extraction yields equivalent to 1 ppb life detection (*i.e.,* 2 pg of DNA from 10^4^ cells in 50 mg of soil). However, nucleic acid–based life detection on Mars may only be practical in the context of extant or recently dead cells as extracellular DNA preserved in soil may be destroyed during sample preparation. Furthermore, we have also demonstrated low-input nanopore sequencing from 2 pg of purified *B. subtilis* spore DNA simulating an ideal extraction yield from a Mars analog soil equivalent to 1 ppb life detection. This was accomplished by employing a genomic carrier (Lambda) to shuttle low-input amounts of *B. subtilis* DNA through library preparation and sequencing without the need for amplification. From the sequencing results, we were able to unambiguously discriminate between Lambda (carrier), *B. subtilis* (true detection), and contamination reads (human and human microflora). However, the detection of high-quality artifactual reads (HQNRs) not mapping to known life represents a false positive and complicates the identification of an unknown organism. Work is currently underway developing SETG through technology readiness level (TRL) 6, which integrates automated extraction, library preparation, and sequencing (Bhattaru, 2018). Validation of the automated system and pending challenges related to the concentration of volumes (*e.g.,* for desalting, reducing elution volume, and library preparation) should soon enable a true sample-to-sequence demonstration. Nevertheless, here we have demonstrated the stepwise process and modifications required for the extraction and sequencing of nucleic acids from low-biomass Mars analog soils. We believe nucleic acids provide a sensitive and unambiguous indicator for life that facilitates distinguishing between forward contamination and putative nucleic acid–based martian life. Long-read nanopore sequencing could conceivably identify extraterrestrial sequences containing nonstandard bases and also discern conserved regions that may suggest a Mars-Earth shared ancestry (Isenbarger *et al.,* 2008).

## Notes

Associate Editor: Christopher McKay

## Abbreviations Used

ddPCR: droplet digital polymerase chain reaction
HQNRs: high-quality noise reads
SASPs: small acid-soluble proteins
SETG: Search for Extra-Terrestrial Genomes
